# Supplementary material for: Histone deacetylase HDA-1 modulates mitochondrial stress response and longevity
Source: Nat Commun. 2020 Sep 15;11:4639. doi: 10.1038/s41467-020-18501-w (PMC7493924; doi:10.1038/s41467-020-18501-w)
Supplement: Supplementary file 1 — Supplementary Information [file 41467_2020_18501_MOESM1_ESM.pdf]

Supplementary Information

Histone deacetylase HDA-1 modulates mitochondrial stress response and longevity

Shao L., Peng Q. et al.

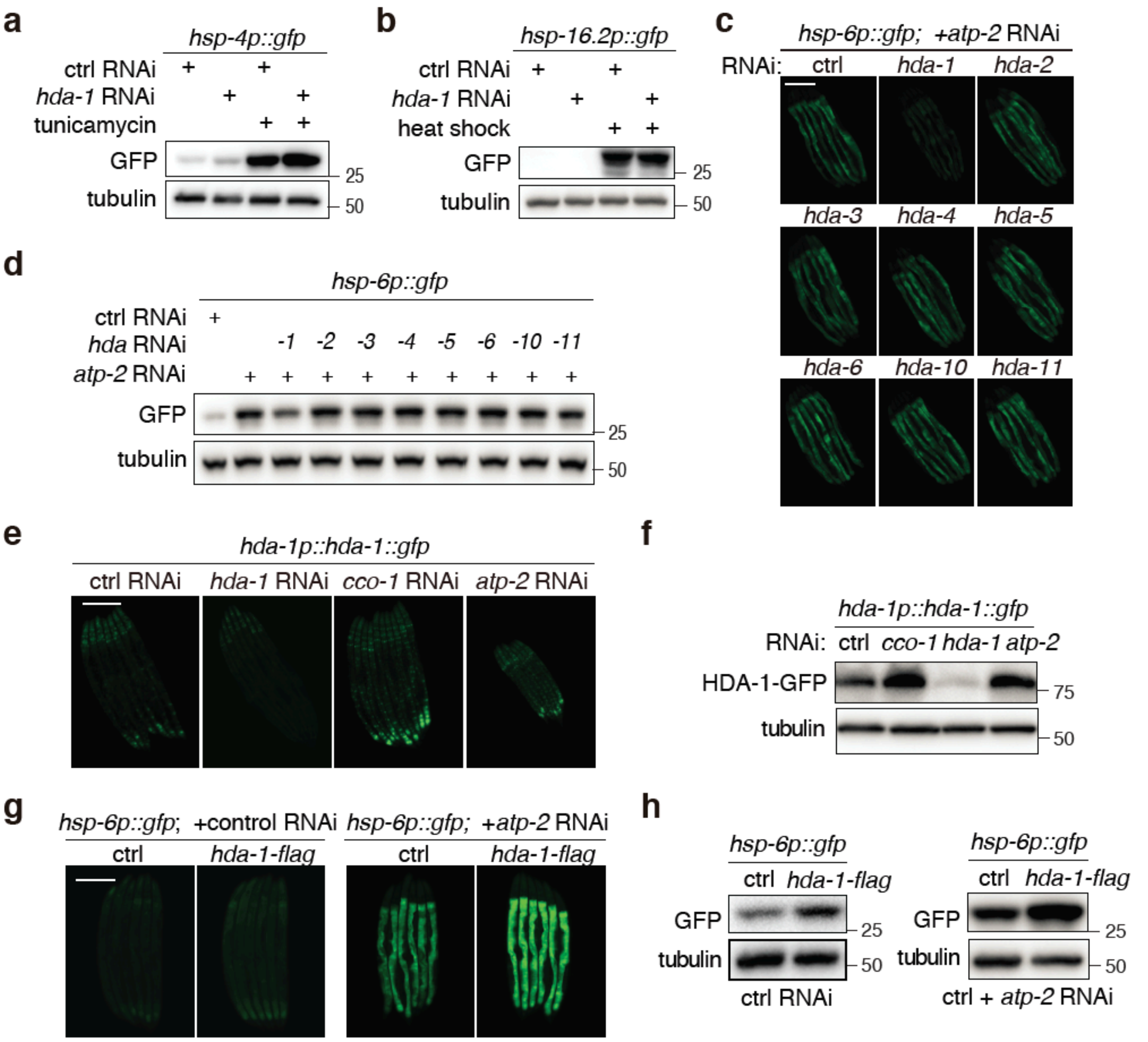

Supplementary Figure 1. HDA-1 is specifically required for UPR^{mt} activation.

a Immunoblotting reveals the GFP expression level in *hsp-4p::gfp* worms fed on control or *hda-1* RNAi, and left untreated or treated with tunicamycin. Tubulin serves as a loading control. **b** Immunoblotting reveals the GFP expression level in *hsp-16.2p::gfp* worms fed on control or *hda-1* RNAi, and left untreated or heat-shocked. Tubulin serves as a loading control. **c, d** Representative fluorescence images (c) and immunoblot analysis (d) of the GFP expression level in *hsp-6p::gfp* worms fed on the indicated *hda* RNAi and treated further with *atp-2* RNAi. Tubulin serves as a loading control. Scale bar, 200 μm. **e, f** Representative fluorescence images (e) and immunoblot analysis (f) of the HDA-1-GFP expression level in *hda-1p::hda-1::gfp* worms fed with control, *hda-1*, *cco-1* or *atp-2* RNAi. Tubulin serves as a loading control. Scale bar, 200 μm. **g, h** Representative fluorescence images (g) and immunoblot analysis (h) of the GFP expression level in *hsp-6p::gfp* worms or *hda-1p::hda-1::flag; hsp-6p::gfp* worms fed with control or *atp-2* RNAi. Tubulin serves as a loading control. Scale bar, 200 μm. Source data are provided as a Source Data file.

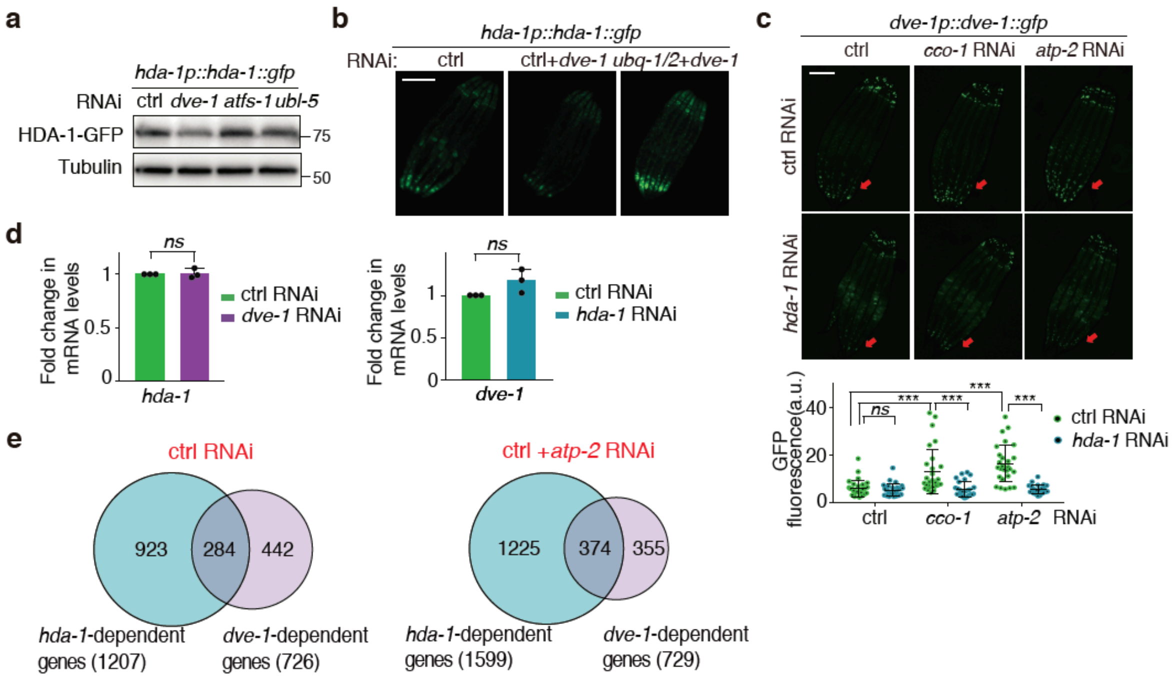

Supplementary Figure 2. HDA-1 and DVE-1 stabilize each other.
a Immunoblotting reveals the GFP expression level in *hda-1p::hda-1::gfp* worms fed on control, *dve-1*, *atfs-1* or *ubl-5* RNAi. Tubulin serves as a loading control. **b** Representative fluorescence images of *hda-1p::hda-1::gfp* worms fed with control RNAi, control+*dve-1* RNAi (1:5 mix) or *ubq-1/2*+*dve-1* RNAi (1:5 mix). Scale bar, 200 μm. **c** Representative fluorescence images of *dve-1p::dve-1::gfp* worms fed with the indicated RNAi. Scale bar, 200 μm. GFP fluorescence was quantified (lower panel). Red arrows indicate the posterior region of the intestine where *dve-1p::dve-1::gfp* is induced or suppressed. n$\approx$30 cells in the posterior region of the intestine per condition. **d** qRT-PCR measurement of the endogenous mRNA levels of the *hda-1* or *dve-1* genes in wild-type worms raised on control RNAi or *dve-1* RNAi, or control RNAi or *hda-1* RNAi (n=3 independent experiments, N $\approx$ 1,000 worms per sample). **e** Venn diagrams comparing *hda-1* (blue) or *dve-1* (purple)-dependent genes in the presence or absence of mitochondrial perturbation. Results in (c) are shown as mean ±SD. P values were calculated by one-way ANOVA and Tukey's multiple comparisons test (ns, no significance; ***P < 0.001). Results in (d) are shown as mean +SD, two-tailed Student's t-test (ns, no significance). Source data are provided as a Source Data file.

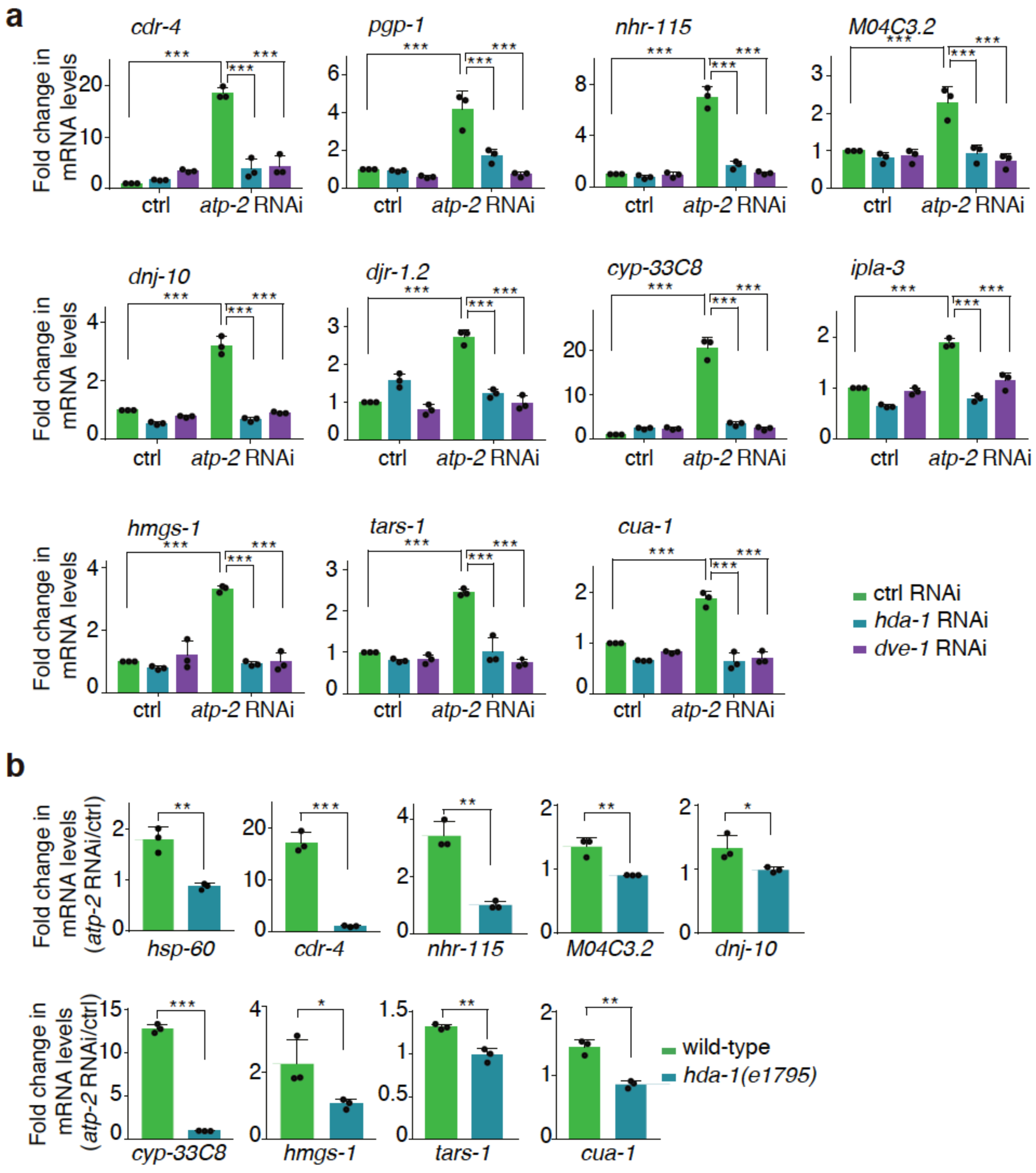

Supplementary Figure 3. The upregulated expression of genes identified in RNA-seq analysis depends on HDA-1 and DVE-1.

a qRT-PCR measurement of the endogenous mRNA levels of the indicated genes in wild-type worms raised on control RNAi, *hda-1* RNAi or *dve-1* RNAi, and untreated or treated with *atp-2* RNAi. **b** qRT-PCR measurement of the endogenous mRNA levels of the indicated genes in wild-type worms or *hda-1(e1795)* mutants. (a, b) n=3 independent experiments, N ≈ 1,000 worms per sample. Results in (a, b) are shown as mean + SD. In (a), P values were calculated by one-way ANOVA and Tukey's multiple comparisons test (***P <0.001). In (b), P values were calculated by two-tailed Student's t-test (*P < 0.05; **P < 0.01; ***P < 0.001). Source data are provided as a Source Data file.

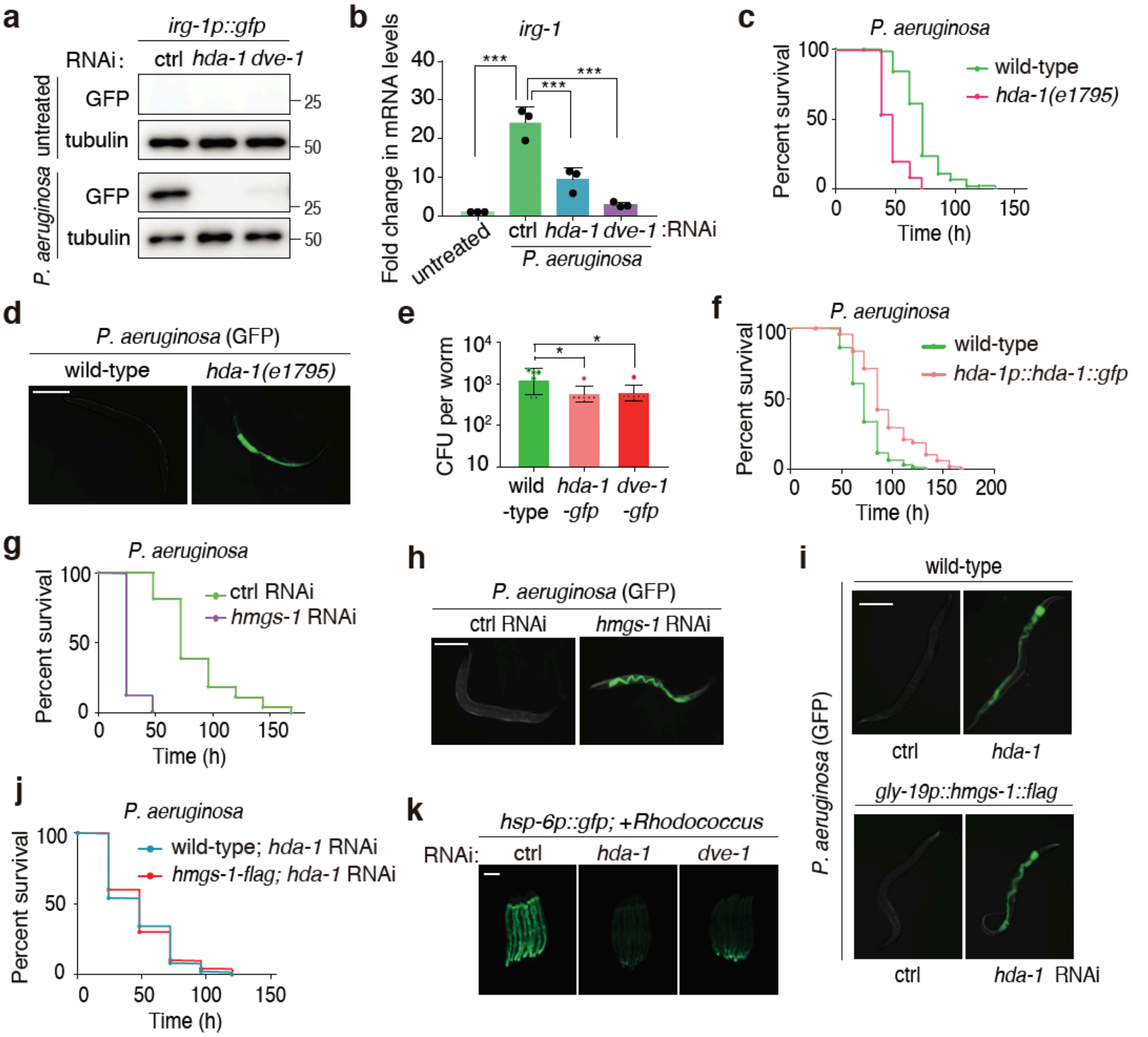

Supplementary Figure 4. HDA-1 and DVE-1 mediate UPR^{mt}-induced innate immune response.

a Immunoblotting of the GFP level in *irg-1p::gfp* worms raised on control, *hda-1* or *dve-1* RNAi and untreated or treated with *P. aeruginosa*. **b** qRT-PCR measures the endogenous *irg-1* mRNA level in wild-type worms raised on control RNAi, *hda-1* RNAi or *dve-1* RNAi and treated with *P. aeruginosa* (n=3 independent experiments, N≈1,000 worms per sample). **c** Survival curves of wild-type or *hda-1(e1795)* mutant worms in the *P. aeruginosa* slow-killing assay. n=65 worms per sample. **d** Representative fluorescence images showing accumulation of *P. aeruginosa* (GFP) in intestines of wild-type or *hda-1(e1795)* mutant worms. Scale bar, 200 μm. **e** CFU (colony forming units) were quantified for *P. aeruginosa* in intestines of wild-type worms, or *hda-1* or *dve-1* overexpression worms. n=30 worms per sample. **f** Survival curves of wild-type worms or *hda-1* overexpression worms in the *P. aeruginosa* slow-killing assay. n=60 worms per sample. **g** Survival curves of wild-type worms treated with control or *hmgs-1* RNAi in the *P. aeruginosa* slow-killing assay. n=55 worms per sample. **h** Representative fluorescence images showing accumulation of *P. aeruginosa* (GFP) in intestines of wild-type worms fed with control or *hmgs-1* RNAi. Scale bar, 200 μm. **i** Representative fluorescence images showing accumulation of *P. aeruginosa* (GFP) in intestines of wild-type or *gly-*

19p::hmgs-1::flag worms fed with control or *hda-1* RNAi. Scale bar, 200 μm. **j** Survival curves of wild-type or *gly-19p::hmgs-1::flag* worms in the *P. aeruginosa* slow-killing assay. n=50 worms per condition. **k** Representative fluorescence images of *hsp-6p::gfp* worms raised on control, *hda-1* or *dve-1* RNAi and treated with *Rhodococcus*. Scale bar, 200 μm. Results in (b) are shown as mean +SD. Results in (e) are shown as mean ±SD. P values were calculated by one-way ANOVA and Tukey's multiple comparisons test (*P < 0.05; ***P < 0.001). Source data are provided as a Source Data file.

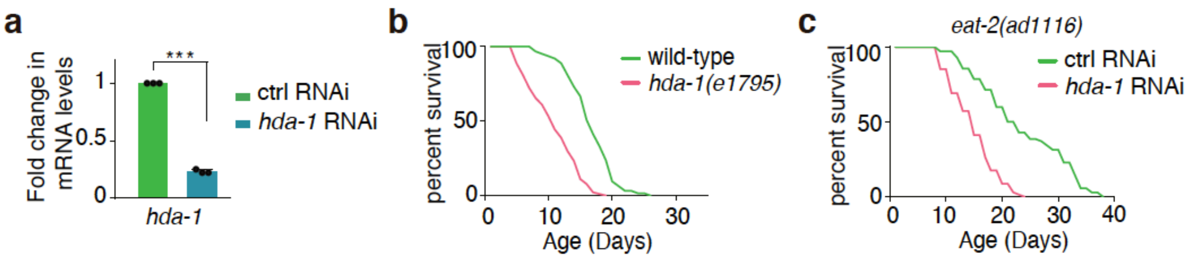

Supplementary Figure 5. Lack of HDA-1 shortens worm lifespan.
a qRT-PCR measurement of the endogenous *hda-1* mRNA level in wild-type worms raised on control RNAi or *hda-1* RNAi (n=3 independent experiments, N≈1,000 worms per sample). **b** Lifespan analysis of wild-type worms or *hda-1(e1795)* mutant worms. n=110 and 100 worms respectively. **c** Lifespan analysis of *eat-2* mutant worms fed with control or *hda-1* RNAi. n=120 worms for each condition. Results in (a) are shown as mean+SD, two-tailed Student's t-test (***$P < 0.001$). Source data are provided as a Source Data file.

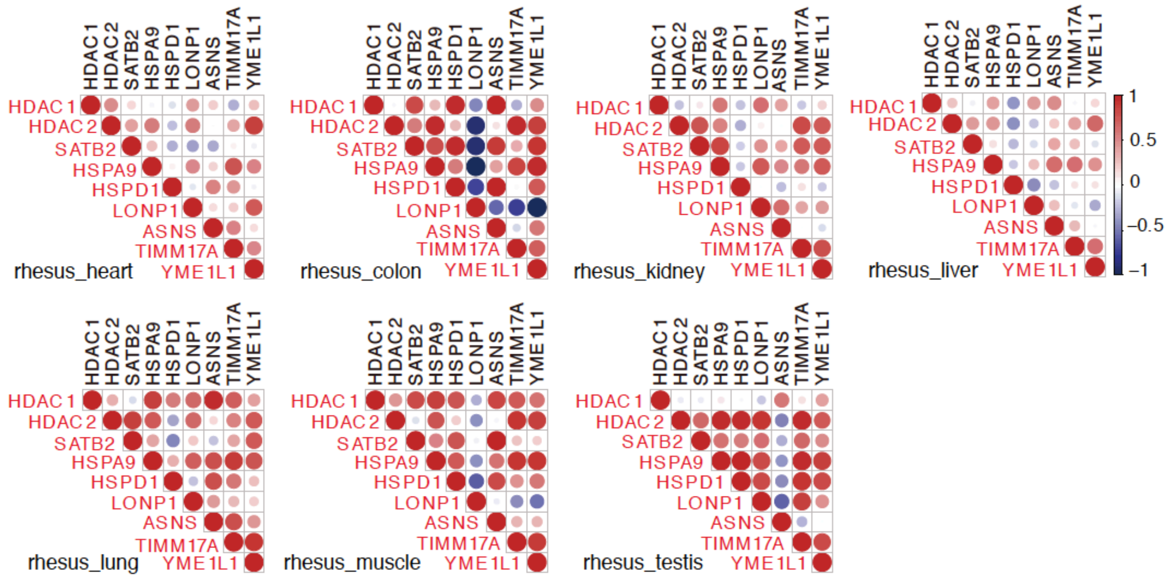

Supplementary Figure 6. Transcript levels of rhesus monkey HDAC1/2 strongly correlate with the expression of UPR^{mt} genes.

Pearson's correlation of HDAC1, HDAC2, SATB2 and UPR^{mt} mRNA levels in rhesus heart, colon, kidney, liver, lung, muscle and testis tissues. Red circles indicate positive correlation and blue circles indicate negative correlation. The size of the circle corresponds to the correlation coefficient. Source data are provided as a Source Data file.

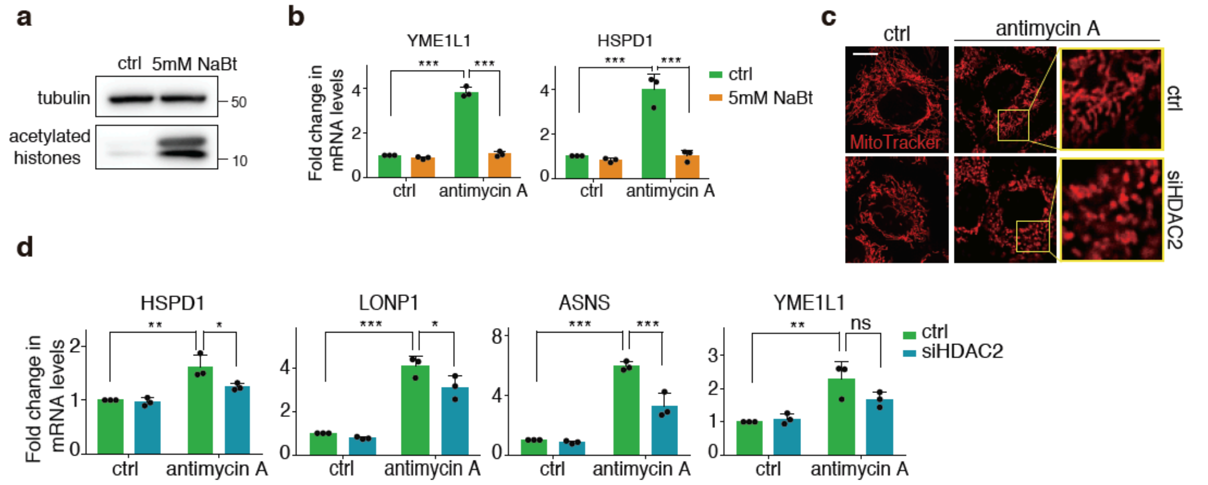

Supplementary Figure 7. The functions of HDAC1/2 and SATB2 in UPR^{mt} activation are conserved in mammals.

a Immunoblotting of acetylated histones in HEK293T cells treated with or without NaBt. Tubulin serves as a loading control. **b** qRT-PCR measures endogenous mRNA levels of YME1L1 and HSPD1 in HEK293T cells cultured with or without NaBt, and followed by Antimycin A treatment. **c** Representative fluorescence images of HeLa cells cultured under the indicated conditions and stained with MitoTracker. Scale bar, 10 μm. **d** qRT-PCR measures endogenous mRNA levels of UPR^{mt} genes in HEK293T cells with or without siHDAC2, and followed by Antimycin A treatment. (b, d) n=3 independent experiments. Results in (b, d) are shown as mean+SD. P values were calculated by two-way ANOVA and Tukey's multiple comparisons test (*P < 0.05; **P < 0.01; ***P < 0.001, ns, not significant). Source data are provided as a Source Data file.

Supplementary Table 1: Primer Sequences used for plasmid construction

	**Primer Sequence (5' – 3')**
hda-1 promoter	CGACCATAGTTGATTACCTACAAAACTAA
	GTTTGATGGTCGCAGTAGACTGA
gly-19 promoter	TTTTTTTCAGTACATTTTTTCATTTC
	CTGGAAATTTAAATTTAATTCTTTG
hda-1	ATGAACTCAAACGGCCCGTT
	CTCTGTCTTCTGACGCTTTTCA
hmgs-1	ATGAGCTTGGGTCAATTGTCATAT
	ATGATGAATTCCGTTCCCGTTTTG
SATB2	ATGGAGCGGCGGAGCGAGAG
	TCTCTGGTCAATTTCGGCAGG

Supplementary Table 2: Primer Sequences used for RT-qPCR

	**Primer Sequence (5' – 3')**
rpl-32	AGGGAATTGATAACCGTGTCCGCA
	TGTAGGACTGCATGAGGAGCATGT
hsp-6	GAGATAAGATCATCGCTGTC
	TCAACTCCTTGCTCCTTCTT
hda-1	GCGTCCGGATTCTGTTACAC
	CTTTCAGGTCTCCGGTTCCT
dve-1	AGCACCCACTCATCATCCAC
	TTGCATCCATGTCGGGTGAG
cdr-4	CAACGGTGAGCATATTGCCG
	CGCTCTTGTAGCGGATGAGA
pgp-1	TCGATTCACCACAGAACGCA
	AGCCATTTGCCAACCGTAGA
nhr-115	TGCAAAACCGACGCAGAAAA
	GCACAGCTGGGCAAACATAA
M04C3.2	GACGGAAAATCCGACGCAAC
	ACTTCTTGGGAAACGGAGGG
dnj-10	GGGTGGAATGGGTGGCTTTA
	ACCACCCTGAAGACGTTGAG
djr-1.2	GTGACCGGAGATGTGTTGGT
	GTTTGCTACATCCGGGTCCA
cyp-33C8	GGACGGCAGGCTTATCTACC
	AGCGGCACAATATTCCCACA
ipla-3	GTGGTAGCCGAGAAGGATCG
	CGTTGGTGAGGGAAGAGAGG
hmgs-1	CACCAGTGACGGATGTTGGA

	GTTGTCCGAGCAGAATCCCA
tars-1	AACGGAGCTGTGTGGGATTT
	GATTGTGCGGTTCTCATGCC
cua-1	CGCAGTGGAACCCCAAAAAG
	TACACACGAAGCACACGTCA
hsp-60	GAGAGAAGAAGGACCGTGTC
	GACTTCATCAATAATCGACGATGG
pals-23	AGGAAACGCTAAGCCAACCA
	AGTTCTGGAGGAACACGAGC
irg-6	AAACCCGCCTCAAGACCTTT
	GCCCGGATAAGCACACTGAA
mul-1	ATACACCTGTGGAACCTGCG
	ACGGTCTGGGAGGAGCTAAT
lys-2	TTCCTCCGTGGATTTGTTCCA
	CCAGTTGGCAGGGGAAGTAA
ACTB	GTCATCACCATTGGCAATGAG
	CGTCATACTCCTGCTTGCTG
HSPD1	CAGTCAAGGCTCCAGGGTTT
	TGGCATCGTCTTTGGTCACA
ASNS	ATCACTGTCGGGATGTACCC
	TGATAAAAGGCAGCCAATCC
LONP1	CAAGCAGACCCACCGTAAGT
	CAGCTCCTCGTCCACAACAT
YME1L1	CCCAGGGACTGGAAAGACAC
	GAGCATTCGCCTTTGCTTCC
HSPA9	TGGTGAGCGACTTGTTGGAAT
	ATTGGAGGCACGGACAATTTT
