# Supplementary material for: Histone deacetylase HDA-1 modulates mitochondrial stress response and longevity
Source: Nat Commun. 2020 Sep 15;11:4639. doi: 10.1038/s41467-020-18501-w (PMC7493924; doi:10.1038/s41467-020-18501-w)
Supplement: Supplementary file 3 — Description of Additional Supplementary Files [file 41467_2020_18501_MOESM3_ESM.docx]

Description of Additional Supplementary Files

File Name: Supplementary Data 1

Description: Differentially expressed genes in *C. elegans*

File Name: Supplementary Data 2

Description: GO annotations of differentially expressed genes in *C. elegans*

File Name: Supplementary Data 3

Description: HDA-1 and DVE-1 ChIP peaks upregulated during mitochondrial stress
