# Supplementary material for: Histone deacetylase HDA-1 modulates mitochondrial stress response and longevity
Source: Nat Commun. 2020 Sep 15;11:4639. doi: 10.1038/s41467-020-18501-w (PMC7493924; doi:10.1038/s41467-020-18501-w)
Supplement: Supplementary file 7 — Reporting Summary [file 41467_2020_18501_MOESM7_ESM.pdf]

nature research

Corresponding author(s): Ying Liu, Chuan-Yun Li

Last updated by author(s): July 24, 2020

Reporting Summary

Nature Research wishes to improve the reproducibility of the work that we publish. This form provides structure for consistency and transparency in reporting. For further information on Nature Research policies, see our Editorial Policies and the Editorial Policy Checklist.

Statistics

For all statistical analyses, confirm that the following items are present in the figure legend, table legend, main text, or Methods section.

n/a	Confirmed	
☐	☒	The exact sample size (*n*) for each experimental group/condition, given as a discrete number and unit of measurement
☐	☒	A statement on whether measurements were taken from distinct samples or whether the same sample was measured repeatedly
☐	☒	The statistical test(s) used AND whether they are one- or two-sided<br>*Only common tests should be described solely by name; describe more complex techniques in the Methods section.*
☒	☐	A description of all covariates tested
☐	☒	A description of any assumptions or corrections, such as tests of normality and adjustment for multiple comparisons
☐	☒	A full description of the statistical parameters including central tendency (e.g. means) or other basic estimates (e.g. regression coefficient) AND variation (e.g. standard deviation) or associated estimates of uncertainty (e.g. confidence intervals)
☐	☒	For null hypothesis testing, the test statistic (e.g. *F*, *t*, *r*) with confidence intervals, effect sizes, degrees of freedom and *P* value noted<br>*Give P values as exact values whenever suitable.*
☒	☐	For Bayesian analysis, information on the choice of priors and Markov chain Monte Carlo settings
☒	☐	For hierarchical and complex designs, identification of the appropriate level for tests and full reporting of outcomes
☐	☒	Estimates of effect sizes (e.g. Cohen's *d*, Pearson's *r*), indicating how they were calculated

Our web collection on statistics for biologists contains articles on many of the points above.

Software and code

Policy information about availability of computer code

Data collection	C. elegans total mRNA reads were generated by sequencing mRNA libraries through Illumina HiSeq 2500.<br>C. elegans DVE-1 and HDA-1 ChIP reads were generated by sequencing ChIP libraries through illumina HiSeq X Ten.
Data analysis	Graph Pad Prism 5.0 for statistics;<br>ImageJ 1.45 for GFP fluorescence quantification;<br>BWA (v0.7.13-r1126) for ChIP-seq reads mapping;<br>Picard (v2.17.6) for removing PCR duplicates;<br>MACS2 (v2.1.1) for peak calling;<br>deep Tools (v2.4.2) for ChIP signal calculation;<br>BEDTools (v2.27.1) for peak overlaps;<br>HOMER (v4.10-0) for motif analysis;<br>Bwtool (v1.0) for peak signal comparison between groups;<br>TopHat2 (v2.1.1) for RNA-seq reads mapping;<br>Cufflinks (v2.2.1) for gene expression level and differentially expressed genes analysis;<br>DAVID (v6.8) for GO term enrichment analysis.

For manuscripts utilizing custom algorithms or software that are central to the research but not yet described in published literature, software must be made available to editors and reviewers. We strongly encourage code deposition in a community repository (e.g. GitHub). See the Nature Research guidelines for submitting code & software for further information.

Data

Policy information about availability of data

All manuscripts must include a data availability statement. This statement should provide the following information, where applicable:

- Accession codes, unique identifiers, or web links for publicly available datasets
- A list of figures that have associated raw data
- A description of any restrictions on data availability

mRNA sequencing data has been deposited in the Gene Expression Omnibus (GEO) database under accession code GSE141041.
ChIP sequencing data has been deposited in GEO database under accession code GSE141042.
Correlation analysis of gene expression levels in different human tissues was performed using data from https://www.gtexportal.org/home/datasets.
Correlation analysis of gene expression levels in different rhesus monkey tissues was performed using data from http://rhesusbase.cbi.pku.edu.cn/download/download.jsp.
Source data for the figures of immunoblotting, lifespan, survival assay, colony-forming units (CFU) quantification, mobility assay have been provided as Source Data files.
All other raw data supporting the findings of this study are available from the corresponding author on reasonable request.

Field-specific reporting

Please select the one below that is the best fit for your research. If you are not sure, read the appropriate sections before making your selection.

☒ Life sciences ☐ Behavioural & social sciences ☐ Ecological, evolutionary & environmental sciences

For a reference copy of the document with all sections, see nature.com/documents/nr-reporting-summary-flat.pdf

Life sciences study design

All studies must disclose on these points even when the disclosure is negative.

Sample size	(1) For fluorescent imaging of worms, sample size was determined according to our pre-tests, >200 worms per sample were sufficient for the experiments.<br>(2) For immunoblotting experiments in worms, sample size was determined according to our pre-tests, >300 worms per sample. For immunoblotting experiments in cells, about 1 million cells per sample. For immunoprecipitation experiments in worms, sample size was determined according to our pre-tests, about 30,000 worms per sample. For immunoprecipitation experiments in cells, cells in one 10cm dish with 90% confluence per sample.<br>(3) For qPCR experiments and mRNA-seq sample preparations, about 1000 worms per sample were used to extract RNA. Sample size was determined according to the published literature (PMID:27400265) and our pre-tests. Sample size was controlled approximately before seeded to plates by counting worms in 3 microliters for 3 times. For RT-qPCR experiments in cells, one well of 12-well plate with about 90% cell confluence per sample. Sample size was determined according to our pre-tests.<br>(4) For ChIP experiments in worms, 240,000 worms per sample. Sample size was determined according to our pre-tests.<br>(5) For lifespan experiment, about 120 worms per experiment. Sample size is determined according to the published literature (PMID:21215371) . For survival assay, about 50 worms per sample. Sample size is determined according to the published literature (PMID:24818940).<br>(6) For mobility assay, about 30 worms per sample were randomly selected and tested under each condition. Sample size was measured under microscope.<br>(7) For colony-forming units quantification, 30 worms per sample. Sample size is determined according to the published literature (PMID:18854822)<br>(8) For expression correlation analyses, gene expression levels in indicated tissue were from GTEx or RhesusBase. All expression data of each gene among all samples were calculated.
Data exclusions	For lifespan measurement, worms that crawled off the plate or underwent vulva blasting that cause irregular death were not included in the data. This exclusion criteria has been established and extensively used in the field.
Replication	All statistical experiments were performed in at least two independent experiments with similar results. Micrographs and Immunoblotting images shown in the figures are representative of three independent experiments with similar results. All attempts at replication were successful.
Randomization	For microscopy, lifespan experiment, survival assay, mobility assay and colony-forming units quantification, samples were randomly selected from culture plates before tests. For immunoblotting, immunoprecipitation, qPCR experiments, mRNA-seq experiments and ChIP experiments, samples under each treatment condition were collected as standard approaches. For expression correlation analyses, all expression levels in each sample were collected from GTEx or RhesusBase.
Blinding	For lifespan experiment, survival assay, mobility assay and colony-forming units quantification, data were collected blindly by technicians without information of the research purpose. For all imaging related experiments, data of different groups were collected and double-checked by the investigators. No blinding was used. For immunoblotting, immunoprecipitation, qPCR experiments, mRNA-seq experiments and ChIP experiments, no blinding was used during data collection. Because samples of different groups were fairly collected and generated simultaneously. For expression correlation analyses, no blinding was used during data collection. Because each expression data is labeled in GTEx or RhesusBase, and the investigators collected all the data belonging to each sample, making blinding not relevant to the study.<br>For all experiments, data were analyzed by softwares or computer codes, the investigators were generally not blinded to group allocation.

Reporting for specific materials, systems and methods

We require information from authors about some types of materials, experimental systems and methods used in many studies. Here, indicate whether each material, system or method listed is relevant to your study. If you are not sure if a list item applies to your research, read the appropriate section before selecting a response.

Materials & experimental systems

n/a	Involved in the study
☐	☒ Antibodies
☐	☒ Eukaryotic cell lines
☒	☐ Palaeontology and archaeology
☐	☒ Animals and other organisms
☒	☐ Human research participants
☒	☐ Clinical data
☒	☐ Dual use research of concern

Methods

n/a	Involved in the study
☐	☒ ChIP-seq
☒	☐ Flow cytometry
☒	☐ MRI-based neuroimaging

Antibodies

Antibodies used

1. Mouse monoclonal anti-GFP antibody: Supplier/Sungene; Cat.No./KM8009; Clone name/9F6; Lot No./H0824.
2. Rat monoclonal anti-tubulin antibody: Supplier/Abcam; Cat. No./ab6161; Clone name/YOL1/34.
3. Rabbit polyclonal anti-FLAG antibody: Supplier/Sigma; Cat. No./F7425.
4. Mouse monoclonal anti-Myc antibody: Supplier/Cell Signaling Technology; Cat. No./2276; Clone name/9B11; Lot No./24.
5. Rabbit polyclonal anti-Acetylated-Lysine antibody: Supplier/Cell Signaling Technology; Cat. No./9441.
6. Rabbit polyclonal anti GFP antibody: Supplier/Abcam; Cat. No./ab290.
7. Mouse monoclonal anti-FLAG magnetic beads: Supplier/Sigma; Cat. No./M8823; Clone name/M2.
8. Mouse monoclonal anti-Myc magnetic beads: Supplier/Bimake; Cat. No./B26302; Clone name is not supplied by the supplier.
9. GFP-trap agarose (coupled with small recombinant alpaca antibody): Supplier/ChromoTek; Cat. No./gta-20; Clone name is not supplied by the supplier.

Validation

All antibodies used in this work were validated by the manufacturers and have been extensive usely in the published studies.
1. Mouse monoclonal anti-GFP antibody: Western blot of C. elegans was validated. Citation [PMID]: 30642431.
2. Rat monoclonal anti-tubulin antibody: Western blots of C. elegans and 293T cells were validated. Citation [PMID]: 30642431,17608927.
3. Rabbit polyclonal anti-FLAG antibody: Western blots of C. elegans and 293T cells were validated. Citation [PMID]: 31645592, 28973854.
4. Mouse monoclonal anti-Myc antibody: Western blot of 293T cells was validated. Citation [PMID]: 22410791.
5. Rabbit polyclonal anti-Acetylated-Lysine antibody: Western blot of 293T cells was validated. Citation [PMID]: 32107550.
6. Rabbit polyclonal anti GFP antibody: Immunoprecipitation of C. elegans was validated. Citation [PMID]: 29463707.
7. Mouse monoclonal anti-FLAG magnetic beads: Immunoprecipitation of C. elegans was validated. Citation [PMID]: 32350248.
8. Mouse monoclonal anti-Myc magnetic beads: Immunoprecipitation of 293T cells was validated. Citation [PMID]: 31091447.
9. GFP-trap agarose (coupled with small recombinant alpaca antibody): ChIP of C. elegans was validated. Citation [PMID]: 29033363.

Eukaryotic cell lines

Policy information about cell lines

Cell line source(s)

HEK293T cells and HeLa cells were purchased from ATCC.

Authentication

Cell lines served in this study were not authenticated right before usage.

Mycoplasma contamination

Cell lines were validated to be free of mycoplasma contamination.

Commonly misidentified lines
(See ICLAC register)

No commonly misidentified cell lines were used.

Animals and other organisms

Policy information about studies involving animals; ARRIVE guidelines recommended for reporting animal research

Laboratory animals

SJ4100(zcIs13[hsp-6::gfp]), SJ4058(zcIs9[hsp-60p::gfp]), CL2070 (dvIs70[hsp-16.2::gfp]), SJ4005(zcIs4[hsp-4p::gfp]), CB5535(hda-1 (e1795)), SJ4197(zcIs39[dve-1p::dve-1::gfp]), AU133(agIs17[irg-1p::gfp]), AM140(rmIs132 [unc-54p::Q35::yfp]), SS104(glp-4(bn2)) and N2 wild-type C. elegans were obtained from the Caenorhabditis Genetics Center (CGC).
The following strains were generated in our lab: YSL1(liuIs1[hda-1p::hda-1::flag; odr-1p::dsRed]), YSL2(liuIs2[hda-1p::hda-1::gfp; odr-1p::dsRed]), YSL3(liuIs1[hda-1p::hda-1::flag; odr-1p::dsRed]; zcIs39[dve-1p::dve-1::gfp]), YSL4(zcIs39[dve-1p::dve-1::gfp]; liuEx1 [hda-1p::hda-1::mCherry; odr-1p::dsRed]), YSL5(liuIs1[hda-1p::hda-1::flag; odr-1p::dsRed]; zcIs13[hsp-6p::gfp]), YSL6(glp-4(bn2); liuIs2[hda-1p::hda-1::gfp; odr-1p::dsRed]), YSL7(glp-4(bn2); zcIs39[dve-1p::dve-1::gfp]), YSL8(glp-4(bn2); rmIs132 [unc-54p::Q35::yfp]), YSL9(glp-4(bn2); liuIs1[hda-1p::hda-1::flag; odr-1p::dsRed]; rmIs132 [unc-54p::Q35::yfp]), YSL10(glp-4(bn2);

	zcIs39[dve-1p::dve-1::gfp]; rmIs132 [unc-54p::Q35::yfp]) and YSL11(glp-4(bn2); liuEx2[gly-19p::hmgs-1::flag; mec-7p::rfp]). All hermaphrodites were tested at late L4 or adulthood stage.
Wild animals	This study did not involve wild animals.
Field-collected samples	This study did not involve samples collected from the field.
Ethics oversight	No ethical approval or guidance was required because we only did research on Caenorhabditis elegans and regular cultured cells.

Note that full information on the approval of the study protocol must also be provided in the manuscript.

ChIP-seq

Data deposition

☒ Confirm that both raw and final processed data have been deposited in a public database such as GEO.

☒ Confirm that you have deposited or provided access to graph files (e.g. BED files) for the called peaks.

Data access links
May remain private before publication.

https://www.ncbi.nlm.nih.gov/geo/query/acc.cgi?acc=%20GSE141042

Files in database submission

HDA-1_input (L4440) rep1
HDA-1_input (L4440) rep2
HDA-1_input (L4440+atp-2) rep1
HDA-1_input (L4440+atp-2) rep2
HDA-1_ChIP (L4440) rep1
HDA-1_ChIP (L4440) rep2
HDA-1_CHIP (L4440+atp-2) rep1
HDA-1_CHIP (L4440+atp-2) rep2
DVE-1_input (L4440) rep1
DVE-1_input (L4440) rep2
DVE-1_input (L4440+atp-2) rep1
DVE-1_input (L4440+atp-2) rep2
DVE-1_ChIP (L4440) rep1
DVE-1_ChIP (L4440) rep2
DVE-1_CHIP (L4440+atp-2) rep1
DVE-1_CHIP (L4440+atp-2) rep2
GSE141042_DG-L.repOverlap.finalPeak.bed.gz
GSE141042_DG-LA.repOverlap.finalPeak.bed.gz
GSE141042_HG-L.repOverlap.finalPeak.bed.gz
GSE141042_HG-LA.repOverlap.finalPeak.bed.gz
DVE-1_input (hda-1) rep1
DVE-1_input (hda-1) rep2
DVE-1_input (hda-1+atp-2) rep1
DVE-1_input (hda-1+atp-2) rep2
DVE-1_ChIP (hda-1) rep1
DVE-1_ChIP (hda-1) rep2
DVE-1_ChIP (hda-1+atp-2) rep1
DVE-1_ChIP (hda-1+atp-2) rep2
HDA-1_input (dve-1) rep1
HDA-1_input (dve-1) rep2
HDA-1_input (dve-1) rep3
HDA-1_input (dve-1+atp-2) rep1
HDA-1_input (dve-1+atp-2) rep2
HDA-1_input (dve-1+atp-2) rep3
HDA-1_ChIP (dve-1) rep1
HDA-1_ChIP (dve-1) rep2
HDA-1_ChIP (dve-1) rep3
HDA-1_ChIP (dve-1+atp-2) rep1
HDA-1_ChIP (dve-1+atp-2) rep2
HDA-1_ChIP (dve-1+atp-2) rep3
HG-D.repOverlap.finalPeak.bed3
HG-DA.repOverlap.finalPeak.bed3
DG-H.repOverlap.finalPeak.bed3
DG-HA.repOverlap.finalPeak.bed3

Genome browser session
(e.g. UCSC)

No longer applicable.

Methodology

Replicates	ChIP-seq was performed by at least two independent biological replicates.

Sequencing depth	>13M 150 bp paired-end reads for each sample; ~80% uniquely mapped reads.
Antibodies	Anti-GFP agarose (GFP-trap agarose). Supplier/ChromoTek; Cat No./gta-20; Clone Name/ NO, it consists of a GFP Nanobody/ VHH coupled to agarose beads.; Lot No./NO. GFP-ChIP experiments were performed in a long range time, so several Lots of GFP-trap agarose were used, but the experiments results are robust.
Peak calling parameters	Reads were mapped to the C. elegans genome (ce11) using BWA(v0.7.13-r1126) with default parameters.<br>MACS2 (v2.1.1) was used to obtain peak regions with following parameter: -g ce -f BAMPE --broad --SPMR.
Data quality	Adaptor sequences were filtered out of reads using an in-house script, then Trim Galore was used to do the quality control with the following parameters: -q 20 --fastqc --illumina --stringency 6 -e 0 --length 50 --trim-n --paired. Processed reads were mapped to the C. elegans genome (ce11) using BWA (v0.7.13-r1126) with default parameters. Only uniquely mapping reads that were properly paired with mapping quality !20 and mismatches <8 were retained. PCR duplicates were removed by Picard-2.17.6 MarkDuplicates command. Qvalue cutoff for narrow/strong and broad/weak regions are 0.05 and 0.01 in peak calling process using MACS2 (v2.1.1) broad mode.
Software	BWA (v0.7.13-r1126) for ChIP-seq reads mapping;<br>Picard (v2.17.6) for removing PCR duplicates;<br>MACS2 (v2.1.1) for peak calling;<br>deepTools (v2.4.2) for ChIP signal calculation;<br>BEDTools (v2.27.1) for peak overlaps;<br>HOMER (v4.10-0) for motif analysis;<br>Bwtool (v1.0) for peak signal comparison between groups;
