# Supplementary material for: Histone deacetylase HDA-1 modulates mitochondrial stress response and longevity
Source: Nat Commun. 2020 Sep 15;11:4639. doi: 10.1038/s41467-020-18501-w (PMC7493924; doi:10.1038/s41467-020-18501-w)

Blots, as shown below, some were cut into slices before incubation with primary antibodies.
The Precision Plus ProteinTM Dual Color Standards, Cat#161-0374,
from BioRad was used as the protein marker.

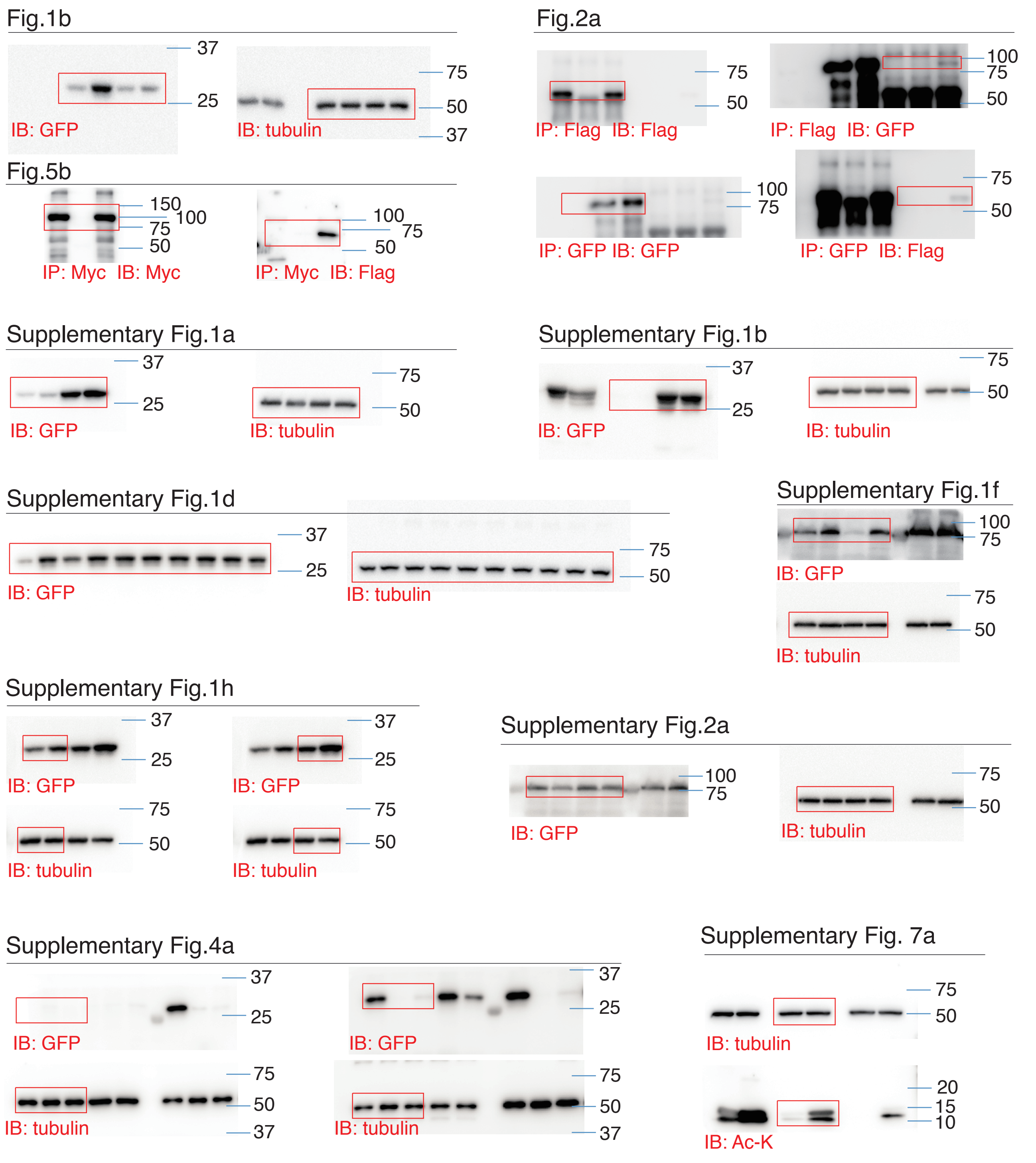

Supplement: Supplementary file 8 — Source Data [file 41467_2020_18501_MOESM8_ESM.zip › Source data/Source data-western blots.pdf]
